# Discontinuous Splenogonadal Fusion: A Case Report

**DOI:** 10.7759/cureus.84795

**Published:** 2025-05-25

**Authors:** Furqan J Al-Bdairi, Kaswer M Altoriahi, Aseel A Alqzweni, Jasim A Almayali, Maytham A Kraidi

**Affiliations:** 1 Department of Laboratory Medicine, Al-Sader Medical City, Najaf, IRQ; 2 Department of Pathology and Forensic Medicine, College of Medicine, University of Kufa, Najaf, IRQ; 3 Department of Urology, College of Medicine, University of Kufa, Najaf, IRQ; 4 Department of Laboratory Medicine, Al-Hussein Teaching Hospital, Samawah, IRQ

**Keywords:** ectopic spleen, orchiectomy, splenogonadal fusion, testicular mass, testis

## Abstract

Splenogonadal fusion (SGF) is a rare congenital anomaly in which accessory splenic tissue fuses with the testis during gestation. SGF is classified into continuous and discontinuous types, depending on the presence or absence of an anatomical connection between the native spleen and the gonads. Due to its uncommon presentation, SGF can easily mislead clinicians, potentially resulting in unnecessary orchiectomy. In this case report, a 27-year-old male presented with a progressive, painless left scrotal mass for one year, noted on self-examination, and had a history of primary infertility and varicocele. A left orchiectomy was performed, and histopathologic examination confirmed the discontinuous type of SGF.

## Introduction

Splenogonadal fusion (SGF), an exceptionally rare congenital abnormality, is a unique and intriguing condition involving an abnormal association between splenic and gonadal mesonephric remnants [1]. First reported by Bostroem in 1883, its classification into continuous and discontinuous types was later described by Putschar WG and Manion WC in 1956 [2,3]. The continuous type features a direct connection between the native spleen and the gonadal structure, whereas the discontinuous type lacks this anatomical link.

The continuous type of SGF has been associated with other congenital anomalies, including cardiac defects, micrognathia, hypoglossia, and cleft palate. In contrast, the discontinuous type is rarely associated with additional congenital abnormalities [4,5].

SGF is most commonly discovered incidentally during surgical exploration for an undescended testis or hernia [6]. While most patients are asymptomatic, some may present with a scrotal mass or symptoms mimicking testicular torsion or epididymo-orchitis [7]. If not carefully considered, SGF may be misdiagnosed as a malignant testicular tumor, potentially resulting in unnecessary, life-altering orchiectomy. This risk underscores the importance of accurate diagnosis and the need to include SGF in the differential diagnosis of scrotal masses, particularly when imaging suggests the presence of splenic tissue.

## Case presentation

A 27-year-old male presented to the urology outpatient clinic with a one-year history of a progressive, painless left scrotal mass detected during self-examination. On palpation, a large, non-tender mass was noted in the left testis, while the right testis was normal. The patient, married for ten years without children, had no history of scrotal surgery. Semen analysis revealed azoospermia.

The patient’s serum alpha-fetoprotein, beta-human chorionic gonadotropin, and lactate dehydrogenase levels were within normal limits (Table 1).

**Table 1:** Patient's serum tumor marker levels.

Serum tumor marker	Patient's serum level	Normal range
Alpha-fetoprotein	3.1 ng/mL	< 10 ng/mL
Beta-human chorionic gonadotropin	0.3 mIU/mL	< 5 mIU/mL
Lactate dehydrogenase	169 U/L	140-280 U/L

**Figure 1:**
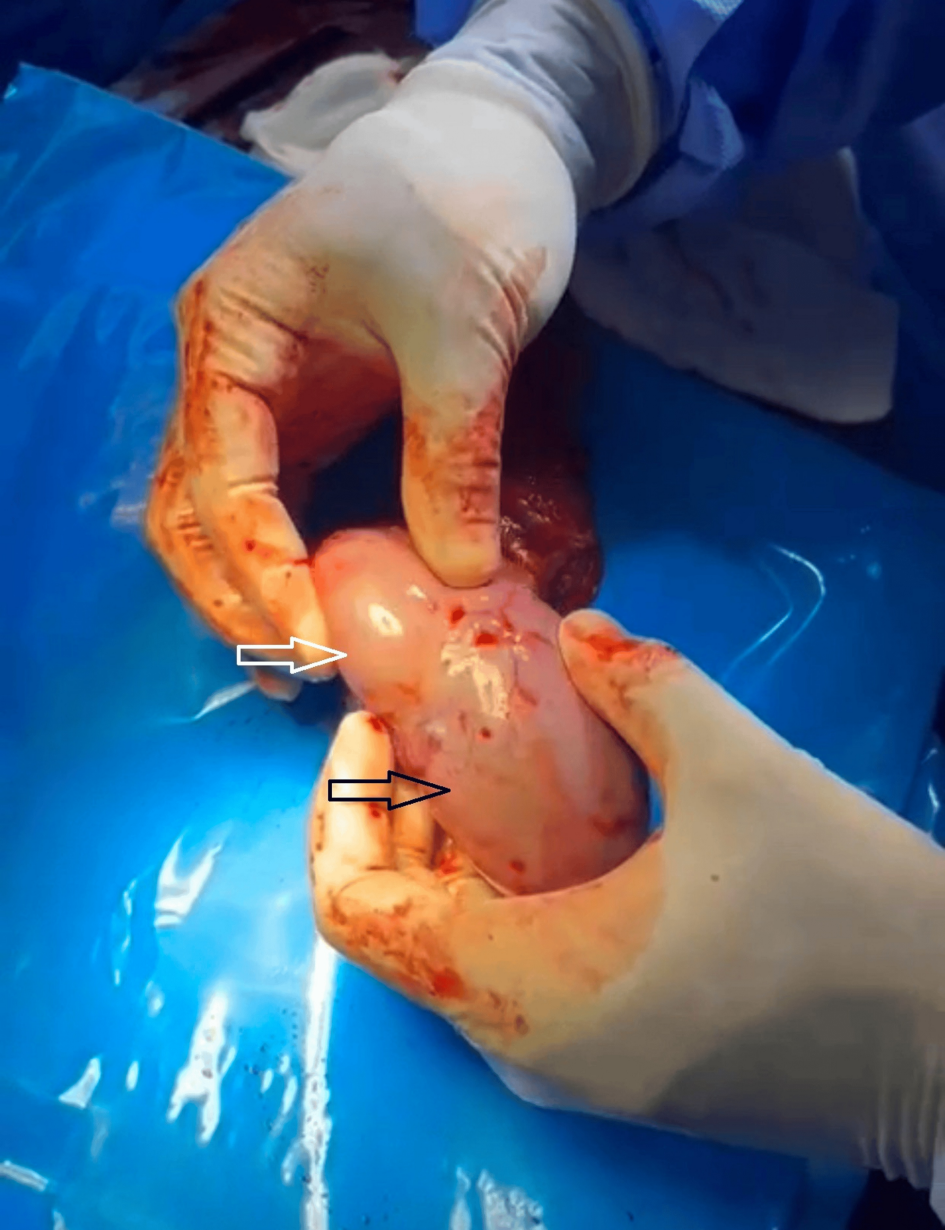
Intraoperative findings showing the spleen (black arrow) and the testis (white arrow).

Colour Doppler ultrasound demonstrated branching vascular flow within the mass. The remainder of the testicular sonogram showed homogenous parenchyma with a grade II left-sided varicocele. These findings were suggestive of seminoma or lymphoma. The case was discussed at a regional multidisciplinary team meeting; however, due to diagnostic uncertainty, the decision was made to proceed with a radical orchiectomy. A left radical orchiectomy was subsequently performed. Intraoperatively, a large mass was identified at the lower pole of the testis (Figure 1).

**Figure 2:**
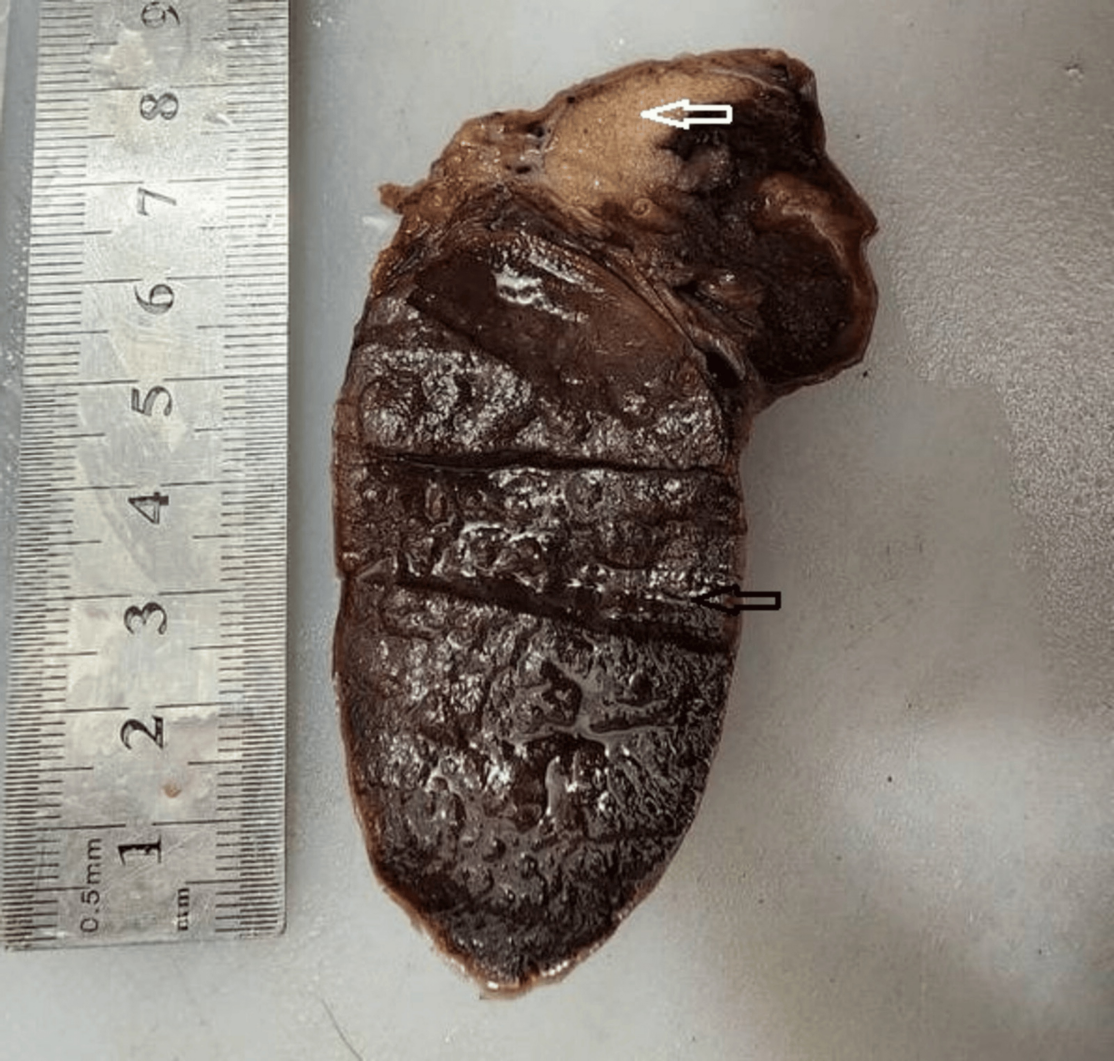
The gross specimen shows an accessory spleen (black arrow) located at the lower pole of the testis (white arrow).

**Figure 3:**
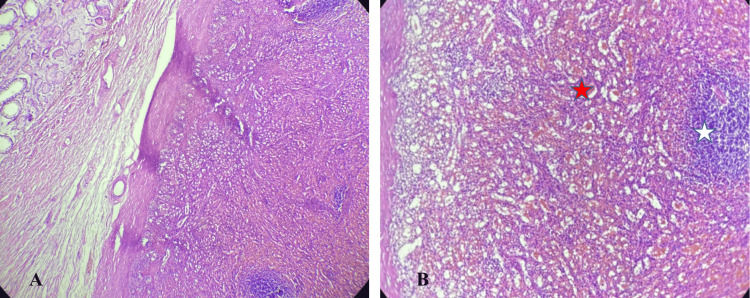
(A) Microscopic appearance shows a distinct fibrous capsule separating the splenic tissue on the right from the testicular tissue on the left (H&E, 40×). (B) The accessory splenic tissue consists of red pulp (red star) with medullary sinuses and white pulp (white star) (H&E, 100×).

**Figure 4:**
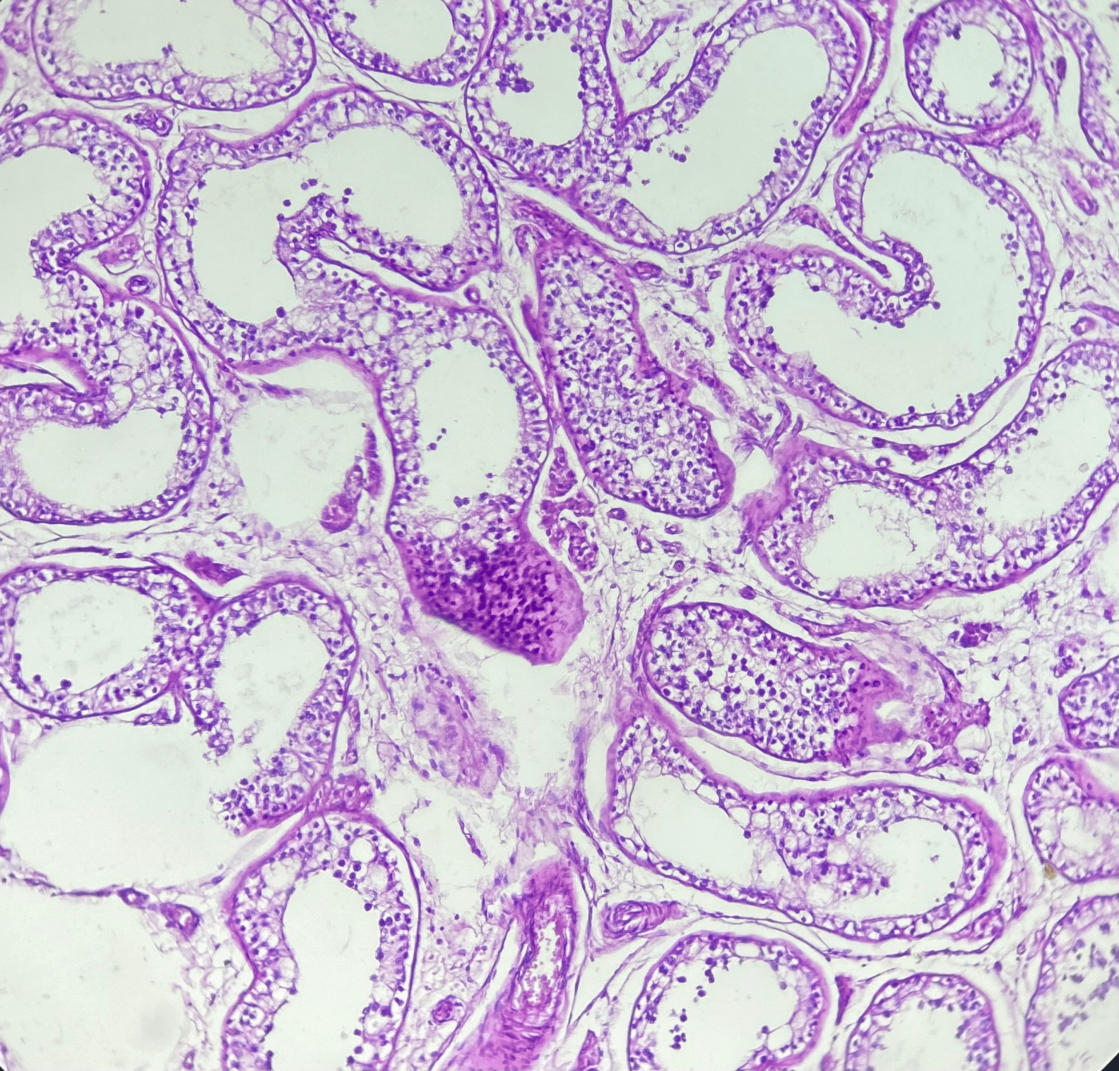
The seminiferous tubules contained Sertoli cells and a reduced number of spermatogenic cells (H&E, 100×).

Gross examination revealed a dark-brown, soft nodule measuring 7 cm in greatest dimension, surrounded by a thick fibrous capsule (Figure 2). Adjacent unremarkable testicular parenchyma and a congested spermatic cord were also noted. Histological examination confirmed the presence of accessory splenic tissue with characteristic native splenic architecture, including well-defined red and white pulp, medullary sinuses, and cords (Figure 3). The testis was distinctly separated from the accessory spleen. It showed histologic features of azoospermia, including seminiferous tubules containing only supporting (Sertoli) cells and a markedly reduced number of spermatogenic cells. Spermatogonia, primary and secondary spermatocytes, spermatids, and spermatozoa were significantly diminished, while Sertoli cells were increased (Figure 4). The surgical margin showed an intact, congested spermatic cord. These findings were consistent with discontinuous-type splenogonadal fusion, with no evidence of malignancy.

The patient had an excellent recovery following orchiectomy, with no postoperative complications and a return to full health. Given the rarity of SGF, the patient was counseled on the condition's benign nature, reinforcing that no further intervention was needed. 

## Discussion

Despite its rarity, approximately 250 cases of SGF have been documented in the literature [8]. SGF predominantly affects males and is most frequently observed on the left side. In the continuous type of SGF, a distinct cord connects the gonad to the original spleen. This cord may consist of splenic tissue, fibrous material, or intervening nodules of splenic tissue with a fibrous cord [9]. In contrast, the discontinuous type of SGF typically presents as isolated inguinal swellings or a firm scrotal mass without any connection to the normally located splenic tissue [10,11].

The etiology of SGF remains unclear, though several theories have been proposed to explain its origin. One theory suggests that an inflammatory condition of the peritoneum covering the spleen and gonads during embryogenesis, between the fifth and eighth weeks of fetal life, could result in partial adhesion of these organs before they descend into the pelvis. Other hypotheses propose that teratogenic events during embryogenesis may cause SGF along with other congenital anomalies [12,13]. These theories provide a broad framework for understanding the potential developmental mechanisms behind SGF.

Although this malformation can affect both sexes, it is more commonly observed in males. This is likely because the male gonads are located superficially, making them more accessible for clinical examination and detection. In contrast, the deeper anatomical location of female gonads often results in SGF being discovered incidentally during surgery for unrelated conditions or at autopsy [11].

A definitive diagnosis of this rare malformation cannot be established based on radiological findings alone. However, the presence of a well-circumscribed, encapsulated, homogenous hypoechoic nodule adjacent to the testicular tissue, with central hypervascularity and a branching pattern similar to that of normal splenic tissue, along with normal serum testicular tumor markers, should raise suspicion of SGF. Conversely, a disorganized branching vascular pattern from the testicular mass on color Doppler ultrasound may suggest testicular malignancy [14].

In fact, histopathological evaluation of the operative specimen remains the gold standard for confirming the diagnosis of SGF [7]. The accessory spleen exhibits the same essential histological features as native splenic tissue, including a peripheral capsule, cortex, medulla, sinusoids, and both white and red pulp.

Preservation of the testicular tissue is possible, and unnecessary orchiectomy can be avoided if an accurate radiological diagnosis is made. The ectopic splenic tissue can often be carefully separated from the covering tunica albuginea without damaging the testis. However, it has been reported that approximately one-third of SGF cases still require orchiectomy due to the increased risk of malignancy associated with cryptorchidism, testicular atrophy, or inseparable splenic tissue [11,13,15].

## Conclusions

SGF is a rare benign anomaly with ambiguous presentations that can easily mislead clinicians. Awareness of this condition, along with familiarity with its radiological and pathological features, is essential for optimal surgical management and to avoid unnecessary orchiectomy. A combined approach using testicular ultrasound, color Doppler, and pathological examination is crucial, not only to rule out malignancy but also to prevent overtreatment. In most cases, complete excision of the splenic tissue is sufficient, and preserving testicular tissue, particularly in younger patients, should be prioritized.
